# Vaccination and Antiviral Treatment Reduce the Time to Negative SARS-CoV-2 Swab: A Real-Life Study

**DOI:** 10.3390/v15112180

**Published:** 2023-10-30

**Authors:** Andrea De Vito, Giulia Moi, Laura Saderi, Mariangela V. Puci, Agnese Colpani, Laura Firino, Anna Puggioni, Sergio Uzzau, Sergio Babudieri, Giovanni Sotgiu, Giordano Madeddu

**Affiliations:** 1 Unit of Infectious Disease, Department of Medicine, Surgery, and Pharmacy, University of Sassari, 07100 Sassari, Italy; giuliamoi95@gmail.com (G.M.); colpani.agnese@gmail.com (A.C.); babuder@uniss.it (S.B.); giordano@uniss.it (G.M.); 2 Clinical Epidemiology and Medical Statistics Unit, Department of Medicine, Surgery and Pharmacy, University of Sassari, 07100 Sassari, Italy; lsaderi@uniss.it (L.S.); mvpuci@uniss.it (M.V.P.); gsotgiu@uniss.it (G.S.); 3 Division of Microbiology and Virology, Department of Biomedical Sciences, University of Sassari, 07100 Sassari, Italy; l.firino@studenti.uniss.it (L.F.); anna.puggioni@aouss.it (A.P.); uzzau@uniss.it (S.U.)

**Keywords:** negativization, COVID-19, antiviral treatment, vaccination, recovery time, monoclonal antibodies, remdesivir, molnupiravir, nirmatrelvir/ritonavir

## Abstract

Clinical trials demonstrated the role of vaccines and antiviral treatments against SARS-CoV-2 in reducing the likelihood of disease progression and death. However, there are limited data available regarding the time to negativity of people who received these treatments. Further, several comorbidities and risk factors might affect the impact of vaccines and antiviral treatments. To this end, we aimed to evaluate and disentangle the impact of anti-SARS-CoV-2 treatments and that of underlying clinical factors associated with a shortened length of SARS-CoV-2 infection. Hence, we recorded the timeframe of positive nasopharyngeal swab in people infected while being hospitalized for reasons other than SARS-CoV-2 infection. All patients who died or were discharged with a positive swab were excluded from the study. A total of 175 patients were included in this study. Clinical conditions encompass malignancies, immunological disorders, cardiovascular, metabolic, neurodegenerative, and chronic kidney disease. Most of the participants (91.4%) were vaccinated before admission to the hospital, and 65.1% received antiviral treatment within three days after the symptom’s onset. Unvaccinated patients had a longer median time to negativity than people who received at least two doses of vaccine (18 vs. 10 days). Concerning the clinical conditions of all patients, multivariate analysis highlighted a lower probability of 14-day conversion of antigenic test positivity in patients with hematological malignancy, including those vaccinated and those exposed to antiviral therapies. In conclusion, our data showed that prompt administration of antiviral treatments accelerates the clearance of SARS-CoV-2. Further, in the elderly patients under study, previous vaccination and antiviral treatment synergize to reduce time to negativity. This translates into a shorter hospitalization time and a lower risk of transmission through patients and connected healthcare workers in a hospital ward setting, with considerable improvement in cost-effective care management.

## 1. Introduction

At the end of 2019, a new coronavirus was held responsible for a cluster of pneumonia in Wuhan, a city in the Chinese province of Hubei; this cluster spread rapidly, leading first to an epidemic in China and subsequently to a global pandemic. In February 2020, the WHO named the disease resulting from the infection “COVID-19” (COronaVIrus Disease 2019). The virus, first named 2019-nCoV, was subsequently isolated and named SARS-CoV-2, due to the strong resemblance to the SARS-CoV-19 virus [1]. To date, the incidence of SARS-CoV-2 infections is over 650 million globally, with little less than seven million deaths.

COVID-19 can cause several symptoms: cough (50%), fever (43%), myalgia (36%), headache (34%), dyspnea (29%), sore throat (20%), diarrhea (19%), nausea/vomiting (12%), ageusia (<10%), anosmia (<10%), runny nose/nasal congestion (<10%) [2,3,4,5,6].

By the end of 2020, several vaccines had been distributed worldwide with a large preventive impact. At the end of 2021, antiviral therapies [7,8,9] (remdesivir and molnupiravir (both inhibitors of SARS-CoV-2 polymerases), nirmatrelvir/ritonavir (inhibitor of SARS-CoV-2 main protease), and the monoclonal antibodies sotrovimab and casirivimab/imdevimab [10,11] became available worldwide, reducing the disease severity and mortality [12,13,14,15]. Complications in severe COVID-19 cases, such as ARDS (acute respiratory distress syndrome), stroke, and multiple organ failure, are profoundly influenced by the cytokine storm. This hyperactive immune response leads to widespread inflammation and tissue damage, exacerbating respiratory distress and causing detrimental effects on various organ systems, thereby escalating the severity and fatality of the disease [16].

Studies demonstrated the efficacy of vaccines and antivirals in terms of reduction of disease progression and death [17,18,19]. However, poor information is available on the time length of negativization (TLN) in individuals exposed to antiviral drugs. Reduced TLN can impact morbidity, mortality, and direct and indirect costs: longer TLN results in longer respiratory isolation and hospitalization [20,21]. Recognizing the profound impacts of prolonged SARS-CoV-2 positivity on both individual and public health highlights the paramount importance of developing strategies to reduce the TLN. Moreover, the potential synergy between vaccination and antiviral therapies in hastening viral clearance is key to optimizing treatment protocols and minimizing the burden of the disease. Considering the evolving SARS-CoV-2 variants and the ongoing challenges in managing COVID-19, this study aims to contribute valuable insights into how the combined approach of vaccination and antiviral treatment can effectively modify the progress of the infection and improve clinical outcomes in patients with different comorbidities.

The aim of this study was to evaluate the impact of vaccination and antiviral therapies on time to SARS-CoV-2 negativization. In addition, variables associated with 14-day negativization were evaluated.

## 2. Materials and Methods

### 2.1. Patient Recruitment and Management

A retrospective cohort study was conducted. Individuals who acquired SARS-CoV-2 infection while being hospitalized for other reasons at the University Hospital of Sassari (AOUSS) between 1 January 2022, and 31 October 2022 were recruited. According to the AOUSS procedures, all SARS-CoV-2 patients were transferred to restricted areas and underwent testing every 24–48 h to promptly release negative individuals from respiratory isolation; therefore, the TLN was precisely established. Patients who died or were discharged with a positive swab, those with incomplete data, and those aged less than 18 years were excluded.

Prescriptions for antiviral therapies (such as molnupiravir, nirmatrelvir/ritonavir, and remdesivir), as well as monoclonal antibodies (including casirivimab/imdevimab and sotrovimab), followed the guidelines set forth by the Italian Drug Agency. The qualifications for administering these treatments included the recent emergence of symptoms (no more than 5 days for molnupiravir and nirmatrelvir/ritonavir, and no more than 7 days for remdesivir and monoclonal antibodies), absence of a requirement for supplementary oxygen, and a heightened likelihood of illness progression due to the existence of one or more chronic conditions such as: (i) obesity with a body mass index exceeding 30; (ii) diabetes mellitus with associated organ damage or hemoglobin A1c above 7.5%; (iii) renal failure; (iv) acute pulmonary disease; (v) significant heart disease; (vi) immune system disorders; (vii) malignancies. Exclusions encompassed (i) an estimated glomerular filtration rate (eGFR) of under 30 mL/min/1.73 m^2^ (specifically for nirmatrelvir/ritonavir and remdesivir); (ii) gestation; (iii) progressed chronic hepatic disease. Furthermore, males prescribed Molnupiravir had to commit to utilizing condoms for a minimum of three months if partnered with a fertile female, and fertile females had to agree to use condoms for a minimum of four days following the conclusion of treatment.

Regarding treatment, the decision rested with the treating physician if multiple options were appropriate. Factors influencing this decision included potential drug interactions, acute kidney dysfunction, a need for hospitalization, the capacity to ingest orally, and the fragility of the patient.

Early treatment was defined as the start of antiviral treatment within three days since the symptom’s onset.

### 2.2. Endpoints

The main objective of our study was to evaluate the impact of vaccination and antiviral treatment on the time of negativization.

The secondary outcome was to evaluate the 14-day negativization and to assess the impact of the variables on it.

### 2.3. Data Collection

Data on demographics (age, gender, and weight), medical history (including chronic renal disease, dialysis, immunodeficiency, transplantation, rheumatologic disease, diabetes, COPD, hemoglobinopathy, neurological disease, cancer, and cardiovascular disease), vaccination status (number of doses and time since the last dose), symptoms (fever, cough, tachypnea, ageusia, pharyngodynia, chills, asthenia, headache, myalgia, gastrointestinal symptoms, dyspnea, nasal congestion, and anosmia), computer tomography (CT) signs, biochemical indicators at admission (white blood cells -WBC-, neutrophils lymphocyte, neutrophil-lymphocyte-ratio -NLR-, ferritin, procalcitonin -PCT-, urea, creatinine, Estimated Glomerular Filtration Rate -eGFR- calculated with the Cockcroft-Gault formula-, aspartate aminotransferase -AST-, alanine aminotransferase -ALT-, De Ritis ratio -AST/ALT-, lactate dehydrogenase -LDH-, C-reactive protein -CRP-, and D-Dimer), early antiviral therapy [molnupiravir, nirmatrelvir/ritonavir(r), and remdesivir], monoclonal antibodies (casirivimab/imdevimab and sotrovimab), and TLN were collected.

### 2.4. SARS-CoV-2 Detection in Respiratory Specimens

Trained healthcare professionals collected NPS specimens. Samples were consistently collected with Copan FLOQSwabs in 3 mL of Universal Transport Medium (UTM, Copan Italia S.p.A., Brescia, Italy). Methods to detect SARS-CoV-2 infection included molecular and antigenic tests. NPS samples underwent RT-PCR testing with the Seegene Allplex SARS-CoV-2 assay (Seegene Inc., Seoul, Republic of Korea), targeting the envelope (E), RNA-dependent RNA polymerase (RdRP)/S, and nucleocapsid (N) SARS-CoV-2 genes. NPS samples were validated as positive when a diagnostic cycle CT of ≤35 was reported for all the assay’s targets. Antigenic tests were performed with the Standard F200 platform after mixing 350 μL of sample’s UTM with the extraction buffer provided by the manufacturer (SD Biosensor, Seoul, Republic of Korea). According to the AOUSS procedures, negativization was assessed only by antigenic assays.

### 2.5. Ethics

The study was conducted in accordance with the Declaration of Helsinki and approved by the Institutional Ethics Committee with the protocol code PG/2022/20481.

### 2.6. Statistical Analysis

Sample characteristics were described with median and interquartile range (IQR) for quantitative variables (e.g., age) and with absolute and relative (percentages) frequencies for qualitative ones (e.g., vaccination status). To compare the time to SARS-CoV-2 negativity between vaccinated and unvaccinated individuals, we employed the Mann–Whitney test. We evaluated the effectiveness of different antiviral therapies using the Kruskal–Wallis test, followed by Dunn’s test for post-hoc comparisons with a Sidak correction.

To visualize and analyze the time to negativity after 14 days, stratified by vaccination status and antiviral therapies, we utilized the Kaplan–Meier curve and log-rank test. The relationship between clinical characteristics and 14-day negative conversion of nasopharyngeal SARS-CoV-2 antigenic test and the presence of interaction was estimated by Cox regression analysis. Statistical significance was set at p-values of less than 0.05, and data analysis was carried out through STATA (Version 17 StataCorp, College Station, TX, USA).

## 3. Results

A total of 175 patients fulfilled the recruitment criteria and were included in the study. Of these, 97 (55.4%) were male, with a median age of 77 (68–83) years. The most prevalent comorbidity was chronic heart failure (37.1%), followed by neurodevelopmental/neurodegenerative diseases (29.1%), obesity (28.6%), diabetes (26.3%, of which 74% with decompensated diabetes), tumors (24.6%; metastatic in 11.4%) and COPD (21.7%). Most patients (91.4%) were vaccinated with at least two doses before admission to the hospital.

Additionally, most patients (88.6%) were asymptomatic at the moment of SARS-CoV-2 diagnoses; of them, 30 (17.1%) remained asymptomatic. Patients’ prevalent symptoms at the time of evaluation, right after testing positive for SARS-CoV-2 infection, were fever (41.7%), cough (28.6%), and dyspnea (20%). Thirty-one (17.7%) patients showed radiological consolidation and GGO, with pulmonary embolism in three cases (Table 1).

Overall, 114 (65.1%) patients received early antiviral therapies; in particular, 65 (37.1%) received a three-day course of remdesivir, 37 (21.1%) molnupiravir, 12 (6.9%) nirmatrelvir/ritonavir, and 44 (25.1) monoclonal antibodies (sotrovimab or Casirivimab/Imdevimab).

**Figure 1:**
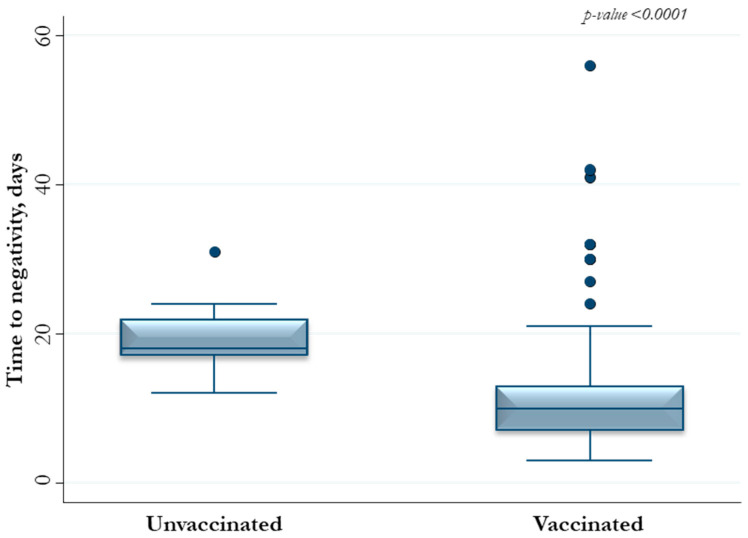
Time length to negativity of 175 people with SARS-CoV-2 positivity according to vaccination status.

**Figure 2:**
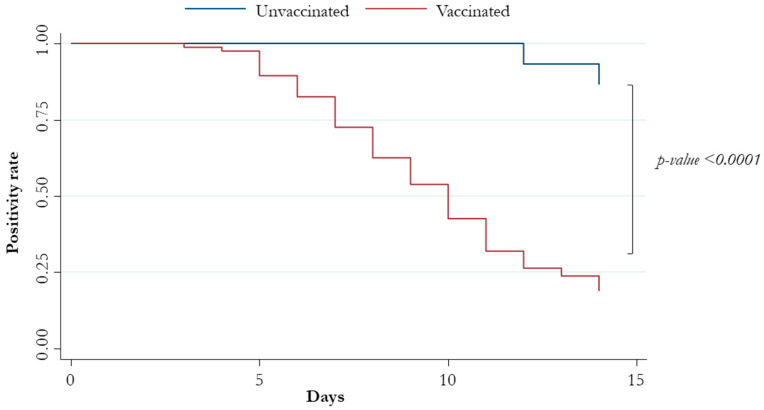
Patients’ negative conversion at 14 days stratified by vaccination status.

Unvaccinated and vaccinated patients with at least two doses showed a median (IQR) time to negativity of 18 (17–22) and 10 (7–13) days, respectively (Figure 1 and Figure 2).

**Figure 3:**
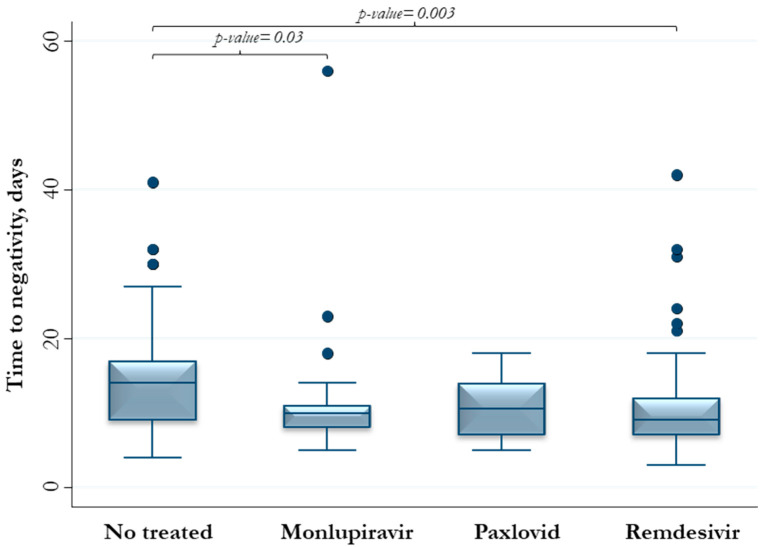
Time length to negativity of 175 people with SARS-CoV-2 positivity according to prescribed therapy.

**Figure 4:**
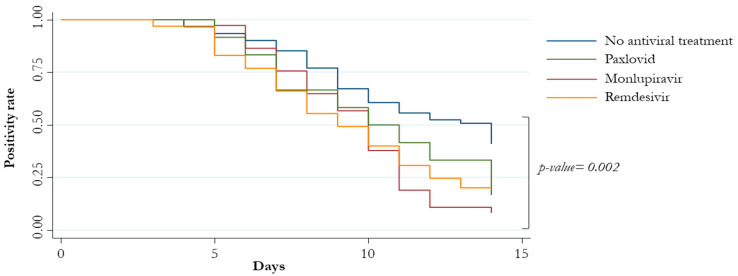
Patients’ negative conversion at 14 days stratified by antiviral therapy.

Patients who did not receive early therapies reported a median (IQR) time to SARS-CoV-2 negativity of 14 days (9–17), whereas those treated with remdesivir, molnupiravir, nirmatrelvir/ritonavir 9 (7–12), 10 (7–14), and 10.5 (7–14) days, respectively (Figure 3 and Figure 4).

**Figure 5:**
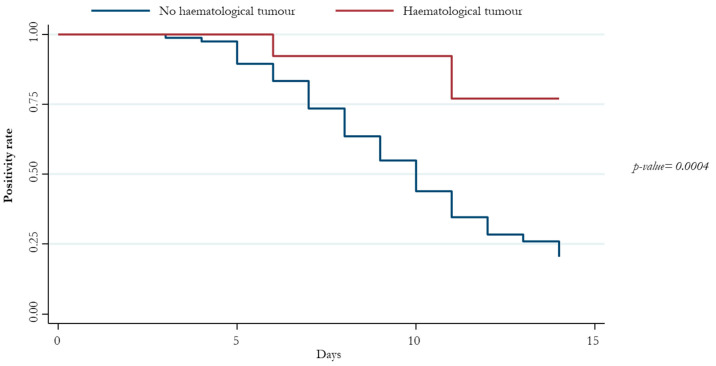
Patients’ negative conversion at 14 days stratified by diagnosis of hematological tumors.

Regarding people with hematological malignancy, we found a significant lower 14-day conversion compared to people without hematological malignancy (Figure 5).

At the multivariate analysis, hematological malignancy was associated with a lower probability of 14-day conversion of antigenic test positivity, whereas those vaccinated and those exposed to antiviral therapies had a higher probability (Table 2).

No relationships were found between biochemical exams and time negativization. The interaction between vaccination and antiviral therapy was not significant (HR = 4.17; 95% CI = 0.25–69.23; *p* = 0.32).

Ten patients were positive after 28 days: Six had hematologic cancers, and four did not receive any therapies (Table 3).

## 4. Discussion

The present study aimed to evaluate the impact of comorbidities, risk factors, vaccination, and antiviral therapies on SARS-CoV-2 negativization in a cohort of patients hospitalized for reasons other than COVID-19. Patients who received at least two doses of vaccine had a shorter time to SARS-CoV-2 negativization when compared with unvaccinated patients. Similarly, exposition to early antiviral treatment significantly shortened the TLN.

Our data are consistent with previous studies reporting the effectiveness of COVID-19 vaccines in reducing the risk of disease severity and hospitalization [22]. Other studies have investigated the potential impact of vaccination in shortening the LTN. Di Lorenzo et al. collected data from 141 cancer patients, showing that vaccinated individuals had shorter TLN than unvaccinated ones (median number of days 12 vs. 20) [23]. Here, we intentionally assessed a cohort with a wider assortment of clinical conditions, including less severe diseases and only 24.6% malignancies, which may partially explain the shorter TLN with a median of 10 days. The impact of vaccination on TLN was also highlighted by Li et al. In their study, the authors showed that TLN was increased according to aging, female gender, fever, cough, and disease severity while consistently shortened according to vaccine doses [24]. Similar findings were reported by Del Borgo et al. in a prospective study conducted in Italy, confirming the efficacy of vaccinations in reducing TLN [25].

Intriguingly, a recent retrospective study found no difference in TLN according to vaccination status in Spanish healthcare workers. Furthermore, in this cohort, previous SARS-CoV-2 infection was associated with a shorter TLN [26]. However, the overall health status of the Spanish healthcare workers is expected to be strikingly different compared to hospitalized patients as those recruited in this study, explaining a less severe infection and a more rapid clearance of the virus. However, the authors do not specify if routine swabs were performed at the time of the study. To this end, the evaluation of vaccine efficacy in the general population is key to controlling SARS-CoV-2 dissemination. Nonetheless, collecting data on the impact of vaccination in patients infected during hospitalization for reasons other than COVID-19 is specifically relevant to predict and control transmission dynamics in hospitalized, fragile populations.

Early treatment has also been reported to reduce the risk of severe disease, hospitalization, and death in patients with COVID-19. A shorter time length of viral shedding may be a further benefit of these drugs, leading to reduced transmission and earlier recovery of the patient. TLN in patients receiving early antivirals has been poorly investigated. An Italian study on nephrological patients described a median negativization time of 31 days. Noteworthy, data were collected between February and April 2020. Available treatments at the time were hydroxychloroquine, darunavir/ritonavir, lopinavir/ritonavir, azithromycin or tocilizumab, and no impact of these drugs on negativization have been reported [27]. Another Italian study supported the role of a three-day course of remdesivir in an outpatient cohort. A shorter median time was described for nirmatrelvir/ritonavir when compared to remdesivir and molnupiravir (8 vs. 10 days). In this study, 230 people received remdesivir three-day course, 499 molnupiravir, and 398 nirmatrelvir/ritonavir [25]. The efficacy of nirmatrelvir/ritonavir was reported in a prospective study where it was compared with sotrovimab. TLN was 11.5 days in the sotrovimab-treated group vs. 4 days in the nirmatrelvir-treated group [28]. Our study found a median negativity time of 9, 10, 10.5 days in patients treated with remdesivir, molnupiravir, and nirmatrelvir/ritonavir.

Cegolon et al., in a recent study, analyzed the effectiveness of Molnupiravir, Nirmatrelvir/Ritonavir, and Sotrovimab in high-risk COVID-19 outpatients. These drugs reduced hospitalizations and death rate but vaccines had a stronger effect on viral clearance [22]. In our study, sotrovimab did not show a significant impact on LTN, probably due to a parallel impact of lack of vaccination that was lower in our cohort (8.6%) compared to the cohort of Cegolon et al. (22.5%) [22].

Regarding the oncohematologic patients, our study highlighted that this population manifests a heightened susceptibility to prolonged SARS-CoV-2 positivity, enduring beyond the 14-day threshold, with a notable subset persisting at 28 days. This observation underscores the necessity for a nuanced therapeutic strategy tailored specifically to this vulnerable cohort. A reconsideration of the current antiviral regimens, such as the exploration of combined antiviral therapies or an extension of the treatment duration, may be warranted to enhance viral clearance efficacy in oncohematologic patients. The implementation of a diversified antiviral approach, potentially incorporating dual antiviral agents or prolonged therapeutic courses, could optimize the management of SARS-CoV-2 infection in these patients, thereby facilitating improved viral negativization rates. Studies about this approach have been conducted. Mikulska et al. studied the virological and clinical response in 22 immunocompromised patients with prolonged/relapsed COVID-19, treated with two antivirals and monoclonal antibodies. The rate of the virological response on day 14 was 75% [29].

Although results are limited by poor sample sizes and because of the observational nature of our study, antiviral therapies and vaccines clearly suggest enabling shortening TLN. Hence, these critical tools may synergically represent real game-changers with an impact on a single patient but also at a community level. The reduction of transmission risk can reduce dissemination in hospital wards but also can shorten individual isolation, with both economic and psychological consequences; a shorter recovery time may also alleviate the burden on healthcare systems and healthcare workers by streamlining hospital bed turnover and improving patient flow [30,31]. However, our data clearly highlight that the management of people with hematological malignancies requires further careful attention, and more efforts are needed to prevent infection and dissemination in the oncology units.

Our study has several limitations that warrant consideration. Firstly, it is a monocentric study, which inherently limits the generalizability of our findings. The sample size is also relatively small, which may affect the robustness of our conclusions. Due to the condition of many patients, obtaining accurate historical infection data was challenging. However, it is important to note that while some patients might have reported previous negative or positive test results, it remains impossible to exclude the possibility of unreported asymptomatic SARS-CoV-2 infections in some individuals. Additionally, the role of tixagevimab/cilgavimab was not evaluated in our study, leaving room for further investigation into the impact of various monoclonal antibodies, especially in immunocompromised patients. Secondly, the role of tixagevimab/cilgavimab was not evaluated. Therefore, the impact of monoclonal antibodies should be further investigated, especially in immunocompromised patients. The strength of our study lies in the precision with which we could determine the timing of SARS-CoV-2 positivity. Knowing the exact moment of positive swabbing for SARS-CoV-2 allowed us to accurately assess the time to viral negativization. This level of precision enhances the reliability of our findings concerning the duration of viral shedding and the effectiveness of various therapeutic interventions. It provides a clear temporal framework within which the impact of vaccines and antiviral therapies on the course of infection could be evaluated, offering valuable insights into their role in managing and controlling the spread of the virus within hospital settings.

## 5. Conclusions

Our data suggest that SARS-CoV-2 vaccines and early antiviral therapies can shorten the duration of viral shedding in hospitalized COVID-19 patients. These findings have implications for individuals and communities. Larger and more heterogeneous populations could prove the reliability of the findings. Finally, further studies should focus on evaluating and refining antiviral combinations and treatment durations specifically for oncohematologic patients, aiming to enhance clinical outcomes and reduce the risks associated with persistent viral presence.

## Figures and Tables

**Table 1:** Characteristics of 175 patients SARS-CoV-2 infected during hospitalization for other reasons.

Variables	Cohort (*n* = 175)
Males, *n* (%)	97 (55.4)
Median (IQR) age, years	77 (68–83)
BMI > 30 kg/m^2^, *n* (%)	50 (28.6)
Chronic renal failure, *n* (%)	28 (16.0)
Immunodeficit, *n* (%)	17 (9.7)
Decompensated diabetes, *n* (%)	34 (19.4)
Diabetes, *n* (%)	46 (26.3)
COPD, *n* (%)	38 (21.7)
Neurodevelopmental/neurodegenerative diseases, *n* (%)	51 (29.1)
Dementia, *n* (%)	28 (16.0)
Cerebrovascular events, *n* (%)	26 (14.9)
Oncological disease, *n* (%)	43 (24.6)
Metastasis, *n* (%)	20 (11.4)
Hematological tumors, *n* (%)	13 (9.4)
Cardiovascular diseases, *n* (%)	69 (39.4)
Heart failure, *n* (%)	65 (37.1)
Previous acute myocardial infarction, *n* (%)	25 (14.3)
Vaccination, *n* (%)	160 (91.4)
Fever, *n* (%)	73 (41.7)
Cough, *n* (%)	50 (28.6)
Pharyngodynia, *n* (%)	19 (10.9)
Headache, *n* (%)	15 (8.6)
Gastrointestinal symptoms, *n* (%)	11 (6.3)
Dyspnea, *n* (%)	35 (20.0)
GGO, *n* (%)	31 (17.7)
Consolidation, *n* (%)	31 (17.7)
Pulmonary embolism, *n* (%)	3 (1.7)
Median (IQR) WBC (×10^3^)	7.1 (5.4–9.4)
Median (IQR) neutrophils	5.3 (3.4–7.7)
Median (IQR) lymphocytes	1.1 (0.8–1.5)
Median (IQR) NLR	4.8 (2.8–8.2)
Median (IQR) ferritin	250 (140–512)
Median (IQR) procalcitonin	0.08 (0.03–0.31)
Median (IQR) urea	35 (25–51)
Median (IQR) AST	21 (16–31)
Median (IQR) ALT	18 (11–28)
Median (IQR) De Ritis	1.1 (0.8–1.6)
Median (IQR) LDH	215 (180–280)
Median (IQR) CRP	4.8 (1.7–10.0)
Median (IQR) D-Dimer	1.4 (0.7–2.5)
Antiviral, *n* (%)	114 (65.1)
Molnupiravir, *n* (%)	37 (21.1)
Nirmatrelvir/ritonavir, *n* (%)	12 (6.9)
Remdesivir (3-day course), *n* (%)	65 (37.1)
Monoclonal antibodies, *n* (%)	44 (25.1)
Casirivimab/Imdevimab, *n* (%)	8 (4.6)
Sotrovimab, *n* (%)	36 (21.2)
Severe disease *, *n* (%)	31 (17.7)
Median (IQR) time to negativity, days	10 (7–14)

BMI: body mass index; COPD: chronic obstructive pulmonary disease; GGO: ground glass opacities; IQR: interquartile range; WBC: white blood cells; NLR: neutrophil-lymphocyte ratio; AST: aspartate aminotransferase; ALT: alanine aminotransferase; LDH: lactate dehydrogenase; CRP: C reactive protein. * Severe disease was defined as the necessity to start oxygen supplementation or increase it if already administered for chronic diseases.

**Table 2:** Cox regression analysis to assess the relationship between clinical characteristics and 14-day conversion of nasopharyngeal SARS-CoV-2 antigenic test.

Variables	Univariate Analysis		Multivariate Analysis	
	HR (95% CI)	*p*-Value	HR (95% CI)	*p*-Value
Males	1.02 (0.72–1.43)	0.93	1.05 (0.73–1.51)	0.79
Age, years	1.00 (0.99–1.01)	0.77	0.99 (0.98–1.00)	0.11
Immunodeficit	0.48 (0.23–0.98)	0.04	0.71 (0.30–1.70)	0.44
Hematological tumors	0.18 (0.06–0.57)	0.03	0.21 (0.06–0.74)	0.02
Vaccination	11.4 (2.82–46.2)	0.001	12.5 (3.05–50.9)	<0.0001
Headache	1.89 (1.08–3.29)	0.03	1.55 (0.85–2.81)	0.15
Exposure to antiviral therapies	1.97 (1.34–2.90)	0.001	2.32 (1.56–3.44)	<0.0001

**Table 3:** Relevant clinical features of patients (*n* = 10) with positive test after 28 days.

Patient	Negativization Time (Days)	Age	Gender	Obesity	COPD	Hematologic Cancer	Vaccination	GGO	Antiviral	Monoclonal Antibodies
1	30.00	78	Female	No	Yes	No	Yes	Yes	No	No
2	56.00	87	Female	No	No	Yes	Yes	No	Molnupiravir	No
3	30.00	79	Female	Yes	No	No	Yes	Yes	No	No
4	30.00	77	Male	No	No	Yes	Yes	No	No	No
5	41.00	87	Female	No	Yes	Yes	Yes	Yes	No	No
6	32.00	78	Female	Yes	No	No	Yes	No	No	No
7	42.00	44	Male	No	No	Yes	Yes	No	Remdesivir	No
8	32.00	89	Female	No	No	No	Yes	No	No	No
9	31.00	78	Male	No	No	Yes	No	No	Remdesivir	Sotrovimab
10	32.00	66	Male	No	No	Yes	Yes	No	Remdesivir	No

COPD: chronic obstructive pulmonary disease; GGO: ground glass opacities.

## Data Availability

Data will be available upon request.
